# Effects of frog skin peptide temporin-1CEa and its analogs on ox-LDL induced macrophage-derived foam cells

**DOI:** 10.3389/fphar.2023.1139532

**Published:** 2023-03-20

**Authors:** Xue-Feng Yang, Xin Liu, Xiao-Yi Yan, De-Jing Shang

**Affiliations:** ^1^ School of Life Science, Liaoning Provincial Key Laboratory of Biotechnology and Drug Discovery, Liaoning Normal University, Dalian, China; ^2^ School of Basic Medical Sciences, Department of Physiology, Jinzhou Medical University, Jinzhou, China

**Keywords:** frog skin peptide, foam cells, atherosclerosis, lipid metabolism, inflammation

## Abstract

**Purpose:** Atherosclerosis is one of the most important pathological foundations of cardiovascular and cerebrovascular diseases with high morbidity and mortality. Studies have shown that macrophages play important roles in lipid accumulation in the vascular wall and thrombosis formation in atherosclerotic plaques. This study aimed to explore the effect of frog skin antimicrobial peptides (AMPs) temporin-1CEa and its analogs on ox-LDL induced macrophage-derived foam cells.

**Methods:** CCK-8, ORO staining, and intracellular cholesterol measurements were used to study cellular activity, lipid droplet formation and cholesterol levels, respectively. ELISA, real-time quantitative PCR, Western blotting and flow cytometry analysis were used to study the expression of inflammatory factors, mRNA and proteins associated with ox-LDL uptake and cholesterol efflux in macrophage-derived foam cells, respectively. Furthermore, the effects of AMPs on inflammation signaling pathways were studied.

**Results:** Frog skin AMPs could significantly increase the cell viability of the ox-LDL-induced foaming macrophages and decrease the formation of intracellular lipid droplets and the levels of total cholesterol and cholesterol ester (CE). Frog skin AMPs inhibited foaming formation by reducing the protein expression of CD36, which regulates ox-LDL uptake but had no effect on the expression of efflux proteins ATP binding cassette subfamily A/G member 1 (ABCA1/ABCG1). Then, decreased mRNA expression of NF-κB and protein expression of *p*-NF-κB p65, *p*-IκB, *p*-JNK, *p*-ERK, *p*-p38 and the release of TNF-α and IL-6 occurred after exposure to the three frog skin AMPs.

**Conclusion:** Frog skin peptide temporin-1CEa and its analogs can improve the ox-LDL induced formation of macrophage-derived foam cells, in addition, inhibit inflammatory cytokine release through inhibiting the NF-κB and MAPK signaling pathways, thereby inhibiting inflammatory responses in atherosclerosis.

## Introduction

Atherosclerosis (AS) is an important risk factor for heart disease, hypertension, and myocardial infarction, and is the main pathological basis of cardiovascular and cerebrovascular diseases (Chen et al., 2020). AS is a complex metabolic disease characterized by the dysfunction of lipid metabolism and chronic inflammation in the intimal space of the vessel (Guo Z. et al., 2022). With the continuous development of anti-atherosclerosis drugs research, cardiovascular protective drugs, antiplatelet drugs, vasoactive drugs and cyclooxygenase inhibitors have been widely used in clinical (Yoshida, 2003). The lipid-regulating drugs for AS, such as HMG-CoA reductase inhibitors, anti-lipid oxidants, and angiotensin-converting enzyme inhibitors, have gradually become the main anti-atherosclerosis drugs (Kahraman et al., 2018).

Antimicrobial peptides (AMPs) are an essential component of the innate immune system. These peptides are encoded by specific genes, which can produce small molecular peptides with biological activity (Zharkova et al., 2019). In recent years, with the increasing research on the anti-inflammatory mechanisms of AMPs, a variety of evidence has suggested that inflammation plays a key role in the pathogenesis of AS, and it is proposed that AMPs play an important role in the treatment of AS inflammation (Kougias et al., 2005). Studies have shown that the antibacterial peptide LL-37 is expressed in human macrophages, and its mRNA expression level increases in AS lesions compared with normal blood vessels (Durr et al., 2006; Edfeldt et al., 2006). PR-39 and LL-37 are both members of the antimicrobial peptide family, and PR-39 has a cardioprotective effect (Bao et al., 2001). In our study, the frog skin peptide temporin-1CEa is isolated and purified from the skin secretion of *Rana chensinensis*. To increase the cationicity of temporin-1CEa, LK2(6) was developed by replacing L-Asp^3^ and L-Gly^16^ with L-Lys. Based on LK2(6), LK2(6)A(L) was designed with an L-Leu substitution for L-Ala^8^ on the hydrophobic surface to increase hydrophobicity. LK2(6) and LK2(6)A(L) exhibited increased net positive charges from +4 to +6, and LK2(6)A(L) had similar hydrophobicity to temporin-1CEa. Previous studies have shown that temporin-1CEa and its analogs exhibit broad-spectrum antimicrobial and antitumor activity. Improve the LPS-stimulated inflammatory environment of mouse macrophages through MyD88-dependent signaling pathways and analogs have a more obvious anti-inflammatory effect compared with temporin-1CEa (Yang et al., 2013; Shang et al., 2014; Shang et al., 2016; Wang et al., 2016; Dong et al., 2017; Wang et al., 2017). Monocyte-derived macrophages play a pivotal role in the lipid metabolism, inflammatory response, and foam cell formation in AS (Vazquez et al., 2020). Macrophages can form foam cells by phagocytosis of endogenous ox-LDL, which initiates early lesion formation in AS (Liu et al., 2022). Ox-LDL can induce macrophages to release inflammatory factors. The macrophage-mediated inflammatory response causes the accumulation of lipids and inflammatory factors, leading to vascular endothelial cell damage in the arterial wall and changes in vascular permeability (Penalver et al., 2020).

Based on this research background, in this study, THP-1 derived human macrophages and murine macrophages RAW264.7 cells were cultured *in vitro*, and ox-LDL was added to establish a macrophage-derived foam cells model. After treatment with the natural AMPs temporin-1CEa, which isolated and purified from the secretions of Chinses brown frog skin and its analogs LK2(6) and LK2(6)A(L), the levels of intracellular lipid droplet, cholesterol, and the release of inflammatory factors were analyzed. Furthermore, the effects of frog skin AMPs on lipid metabolism-related genes, protein expression, NF-κB and MAPK signaling pathway activation in foam cells were studied. The findings of the study will provide a theoretical basis for examining the lipid metabolism and anti-inflammatory mechanism of frog skin peptide temporin-1CEa and its analogs and provide a new therapeutic strategy for the treatment of AS.

## Materials and methods

### Reagents

Ox-LDL was purchased from Yiyuan Biotechnology Co., Ltd. (Guangzhou, China). Frog skin peptide temporin-1CEa and its analogs were synthesized by GL Biochemistry Inc. (Shanghai, China) and the purity of the peptide was greater than 95%.

### Cell culture and proliferation analysis

Human leukemia monocytic cells line THP-1 and murine macrophages RAW264.7 cell were purchased from Jiangsu Kaiji Biology Co., Ltd. (Nanjing, China). Cells were cultured in 1,640 (THP-1) or DMEM (RAW264.7) contains penicillin and streptomycin with 10% FBS, at 37°C in a humidified atmosphere of 5% CO_2_. THP-1 cells were incubated with phorbol 12-myristate 13-acetate (PMA, 100 ng/mL) for 48 h. Then THP-1 cells were adherent to the wall and differentiated into macrophages. Cells were seeded in 96-well plates and cultured for 24 h. Then, the cells were cultured in fresh serum-free 1,640 or DMEM medium for 24 h and treated with ox-LDL at concentrations of 50 μg/mL, 100 μg/mL and 200 μg/mL, respectively. According to previous studies, the concentration of temporin-1CEa and its analogs was selected as 1.56 μM, 3.125 μM and 6.25 μM for THP-1 cells, and 0.937 μM, 1.875 μM and 3.75 μM for RAW264.7 cells, respectively. Then, 10 μL of 5 mg/mL MTT was added to each well. After 4 h, discard the supernatant and 150 μL of DMSO was added to each well. The absorbance of each well was measured with Multiskan FC microplate reader (Thermo Fisher Scientific, USA) at 490 nm.

### Oil red O staining

Lipid droplets in ox-LDL stimulated macrophages were observed by oil red O (ORO) staining (Solarbio, Shanghai, China). Briefly, cells were seeded in 24-well plates, incubated for 24 h, and then treated with ox-LDL and/or temporin-1CEa and its analogs for an additional 24 h. The cell supernatant was removed, and cells in each group were cleaned twice with PBS. The cells were fixed with ORO fixative solution for 20 min then soaked with 60% isopropyl alcohol for 5 min, stained with ORO for 10 min, and then stained with mayer hematoxylin. Images were observed using a light microscope (Leica, GER).

### Cholesterol detection

Macrophages were treated with drugs in each group. Then, the cells were lysed, and the free cholesterol (FC) and total cholesterol (TC) levels in cell lysates were measured using cholesterol detection kits according to the manufacturer’s instructions (Solarbio, Shanghai, China). Cellular protein was measured with a BCA protein assay kit according to the manufacturer’s instructions (Meilunbio, Dalian, China). FC and TC were normalized to cellular protein levels. Cholesterol ester (CE) levels were calculated using the following formula: CE = TC-FC.

### ELISA detection

The levels of inflammatory cytokine TNF-α and IL-6 in the supernatants of THP-1 derived human macrophages and murine macrophages RAW264.7 cells were measured using ELISA kits according to the manufacturer’s instructions (Neobioscience, Shenzhen, China) by a Multiskan FC microplate reader (Thermo Fisher Scientific, USA).

### Flow cytometry detection

Flow cytometry was used to detect the protein expression of CD36 in THP-1 derived foam cells. THP-1 derived human macrophages were seeded in 6-well plates with 1 mL per well and cultured at 37°C in 5% CO_2_. After induced differentiation and drug treatment, the cells were centrifuged at 1,000 rpm/min for 5 min and incubated with 5% BSA at room temperature for 1 h. CD36 antibody (Proteintech, Wuhan, China, 1:500 dilution) was added to THP-1 cells and incubated at room temperature for 2 h. Then fluorescent antibody (Proteintech, Wuhan, China, 1:300 dilution) was added to cells and incubated at room temperature for 1 h. Flow cytometry was performed.

### Quantitative real-time PCR

THP-1 and RAW264.7 cells were treated with drugs in each group. Total RNA was extracted with TRIzol reagent (Invitrogen, Shanghai, China). Reverse transcription reaction was performed using a Super Script™ III kit (Invitrogen, Shanghai, China). Amplification of cDNA was performed in an ABI Prism 7,500 Fast sequence detection system (Applied Biosystems, USA) using an SYBR Premix Ex Taq II kit (TaKaRa, Dalian, China). The primer gene sequences were summarized in Table 1. The relative mRNA expression levels are normalized to that of GAPDH (internal control) by using the Ct values calculated according to the manufacturer’s instructions.

**TABLE 1:** Primer sequences.

Name	Primer sequence	Cell	At (°C)	Extension time s)	NM NO.
CD36	F: 5′-GGCТGTG​ACC​GGA​ACТGTG-3′	THP-1	60	39	NM_000072.3
	R: 5′-AGG​TCТCCA​ACТGGC​ATT​AGA​A-3′				
SR-A1	F: 5′-GCA​GTG​GGA​TCA​CТТTCA​CAA-3′	THP-1	60	39	NM_001363744.1
	R: 5′-AGCТGТCAT​TGA​GCG​AGC​ATC-3′				
ABCA1	F: 5′-ACC​CAC​CCT​ATG​AAC​AAC​ATG​A-3′	THP-1	60	39	NM_005502.4
	R: 5′-GAG​TCG​GGT​AAC​GGA​AAC​AGG-3′				
ABCG1	F: 5′-ATT​CAG​GGA​CCТTTC​CTA​TTC​GG-3′	THP-1	60	39	NM_004915.4
	R: 5′-CTCACCACTATTGAACТ TCCCG-3′				
NF-κB	F: 5′-ATG​TGG​AGA​TCA​TTG​AGC​AGC-3′	THP-1	60	39	NM_001145138.2
	R: 5′-CCT​GGT​CCT​GTG​TAG​CCA​TT-3′				
GAPDH	F: 5′-GGA​GCG​AGA​TCC​CТCCA​AAA​T-3′	THP-1	60	39	NM_001256799.3
	R: 5′-GGCТGTT​GTC​ATA​CTT​CTC​ATG​G-3′				
NF-κB	F: 5′-GGA​GGA​GTC​TGG​TCT​CAG​GAA​GC-3′	RAW264.7	60	39	NM_009045.4
	R: 5′-GGA​CAC​GGT​GCT​ACA​TGC​CTA​TTC-3′				
GAPDH	F: 5′-GCC​AAA​AGG​GTC​ATC​ATC​TC-3′	RAW264.7	60	39	NM_008084.3
	R: 5′-GTA​GAG​GCA​GGG​ATG​ATG​TTC-3′				

AT, annealing temperature.

### Western blotting

The cells treated with drugs in each group were lysed in radioimmunoprecipitation assay (RIPA) lysis buffer. The cellular lysates were separated by 10%–12% SDS–PAGE before being electro-transferred to a PVDF membrane using standard procedures. After being blocked with 5% skim milk in TBST for 1 h at room temperature, the membranes were incubated with specific primary antibodies at 4°C overnight against *p*-NF-κB p65, NF-κB p65 (Cell Signaling Technology, USA, 1:1,000 dilution), *p*-IκB, IκB (Abcam, Shanghai, China, 1:1,000 dilution), *p-*JNK, JNK (Cell Signaling Technology, USA, 1:1,000 dilution), *p-*ERK, ERK (Cell Signaling Technology, USA, 1:1,000 dilution), *p-*p38, p38 (Cell Signaling Technology, USA, 1:1,000 dilution) for THP-1 cells, and CD36 (Cell Signaling Technology, USA, 1:1,000 dilution), SR-A1 (R&D Systems, USA, 1:1,000 dilution), ABCA1 (R&D Systems, USA, 1:1,000 dilution), ABCG1 (Abcam, Shanghai, China, 1:1,000 dilution), *p*-NF-κB p65, NF-κB p65 (Abcam, Shanghai, China, 1:1,000 dilution), *p*-IκB, IκB (Abcam, Shanghai, China, 1:1,000 dilution), *p-*JNK, JNK (Abcam, Shanghai, China, 1:1,000 dilution), *p-*ERK, ERK (Abcam, Shanghai, China, 1:1,000 dilution) and *p-*p38, p38 (Abcam, Shanghai, China, 1:1,000 dilution) for RAW264.7 cells, respectively. Then incubated with anti-rabbit, anti-mouse, or anti-goat IgG antibodies conjugated to HRP. ATP1A1 (Proteintech, Wuhan, China, 1:5,000 dilution) or GAPDH (Proteintech, Wuhan, China, 1:5,000 dilution) was used as an internal control. Bands were visualized by an Azure Biosystems c500 instrument using ECL-Plus detection reagents (Santa Cruz, USA). Densitometric quantification of the protein was performed using ImageJ software.

### Statistical analysis

Statistical evaluation was performed using univariate analysis of variance (ANOVA) to analyze ranked data and a *t*-test to differentiate the means of different groups. The values were considered significant when *p* < 0.05. Data was expressed as mean ± SD and the experiment was repeated thrice to ensure the reproducibility of results. SPSS 18.0 software for Windows (SPSS Inc., Chicago, IL, USA) was used to analyze all data.

## Results

### The effects of frog skin AMPs on cholesterol and lipid droplets in ox-LDL induced foaming cells

**FIGURE 1:**
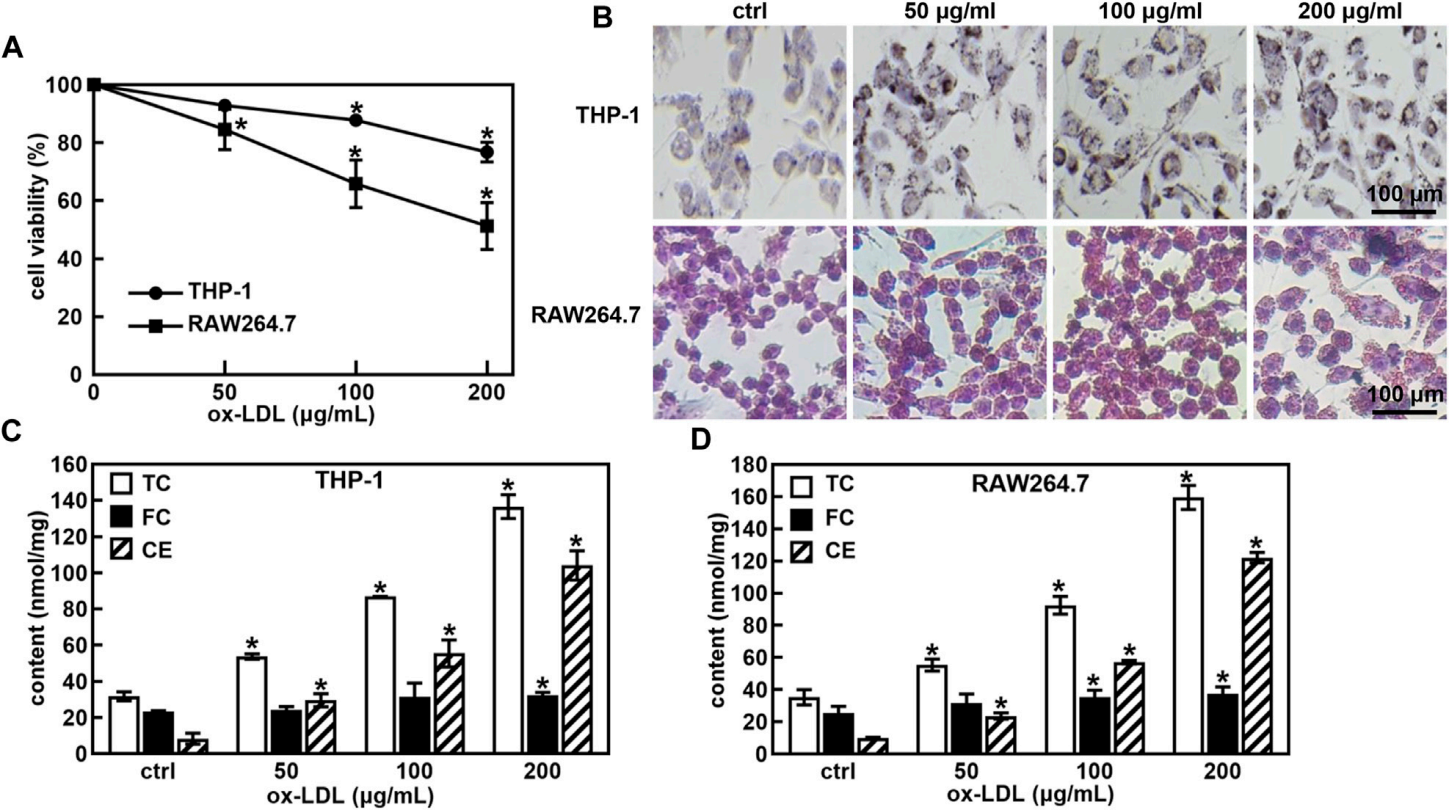
Ox-LDL induced the foaming cell forming of THP-1 and RAW264.7 macrophages. Effects of ox-LDL on the cell viability of THP-1 and RAW264.7 **(A)**; ox-LDL induced cell morphological changes and lipid droplet formation analyzed by ORO staining (**B**, 400X); Levels of total cholesterol (TC), free cholesterol (FC) and cholesterol ester (CE) in ox-LDL induced foaming cells **(C,D)**. *p < 0.05 vs. the control (ctrl) group.

First, the ox-LDL induced macrophage-derived foam cells model was established. A treatment of ox-LDL decreased the cell viability of THP-1 and RAW264.7 cells in a concentration-dependent manner (Figure 1A). Cell viability was approximately 75% and 50% when the ox-LDL concentration was 200 μg/mL in THP-1 and RAW264.7 cells, respectively. The ORO staining results showed that the size and intracellular accumulation of lipid droplets were increased in ox-LDL-induced foaming cells (Figure 1B).

One of the hallmarks of foam cells is that the proportion of cholesterol in the cell is greater than 50% compared to that of normal cells. The TC, FC and CE levels in normal cells and foam cells were measured after the cells were incubated with ox-LDL for 48 h. As shown in Figures 1C, D, the TC and CE levels were significantly increased in a concentration-dose-dependent manner in ox-LDL-induced THP-1 and RAW264.7 cells. When the ox-LDL concentration was more than 100 μg/mL, the proportion of cholesterol in THP-1 and RAW264.7 cells was greater than 50% compared with that in untreated cells, suggesting that 100 μg/mL ox-LDL induced foaming cell formation in THP-1 and RAW264.7 cells.

**FIGURE 2:**
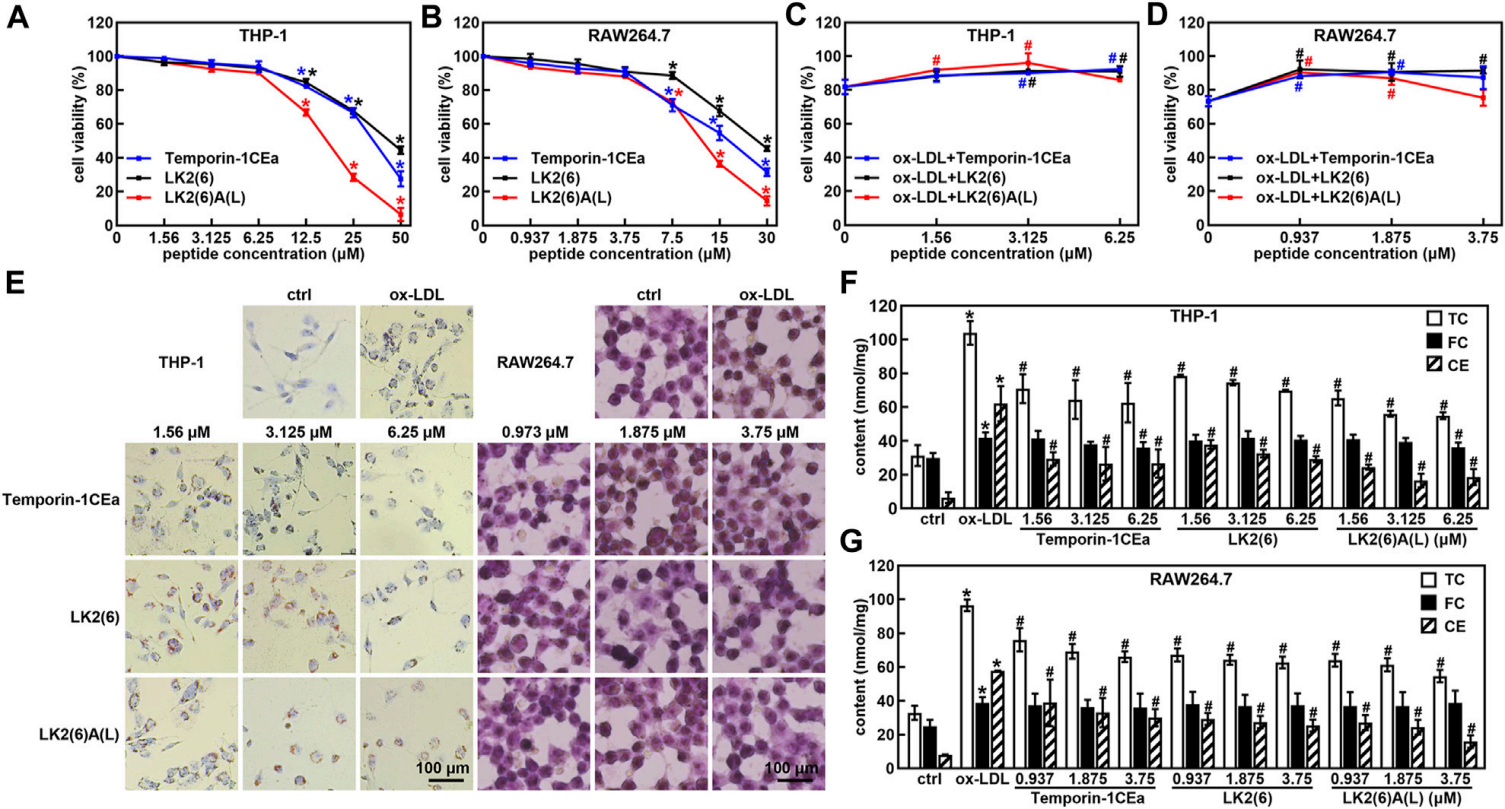
Effects of frog skin AMPs on ox-LDL induced foam cells. Effects of frog skin AMPs on the cell viability of THP-1 cells **(A)**, RAW264.7 cells **(B)** and ox-LDL induced foaming cells **(C,D)**; Cell morphological changes and lipid droplet formation analyzed by ORO staining (**E**, 400X); Levels of total cholesterol (TC), free cholesterol (FC) and cholesterol ester (CE) in the frog skin AMPs treated foam cells **(F,G)**. *p < 0.05 vs. the ctrl group. ^#^ *p* < 0.05 vs. the ox-LDL group.

Subsequently, we examined the effects of three frog skin AMPs at different concentrations on cell viability of THP-1 and RAW264.7 cells. The results showed that the concentration of three frog skin AMPs below 6.25 μM in THP-1 cells and 3.75 μM in RAW264.7 cells did not have significant effects on cell viability (Figures 2A, B). Importantly, three frog skin AMPs significantly increase the cell viability of the ox-LDL-induced foaming macrophages. As shown in Figures 2C, D, compared to the ox-LDL group, frog skin AMPs increased the cell viability by 9.4%–14.2% and 17.1%–18.8% at a concentration of 6.25 μM in THP-1 foam cells and 3.75 μM in RAW264.7 foam cells, respectively, suggesting that temporin-1CEa and its analogs alleviated the damages from ox-LDL on THP-1 and RAW264.7 cells. Temporin-1CEa and its analogs reduced the formation of intracellular lipid droplets (Figure 2E) and decreased the levels of TC and CE in ox-LDL-induced foaming cells, but FC was not significantly altered (Figures 2F, G). Free cholesterol can be converted into cholesterol esters, causing the accumulation of lipid droplets in macrophages. Compared to the ox-LDL group, frog skin AMPs decreased the content of TC in a dose-dependent manner by 32.7%–47.1% and 31.4%–43.3%, and the content of CE by 52.9%–73.2% and 48.0%–72.4% at a concentration of 6.25 μM in foaming THP-1 and RAW264.7 cells, respectively. Among them, LK2(6)A(L) exhibited the best inhibitory effect on the levels of TC and CE in foaming THP-1 and RAW264.7 cells.

### Frog skin AMPs inhibited foaming formation by reducing the uptake of ox-LDL in macrophages

**FIGURE 3:**
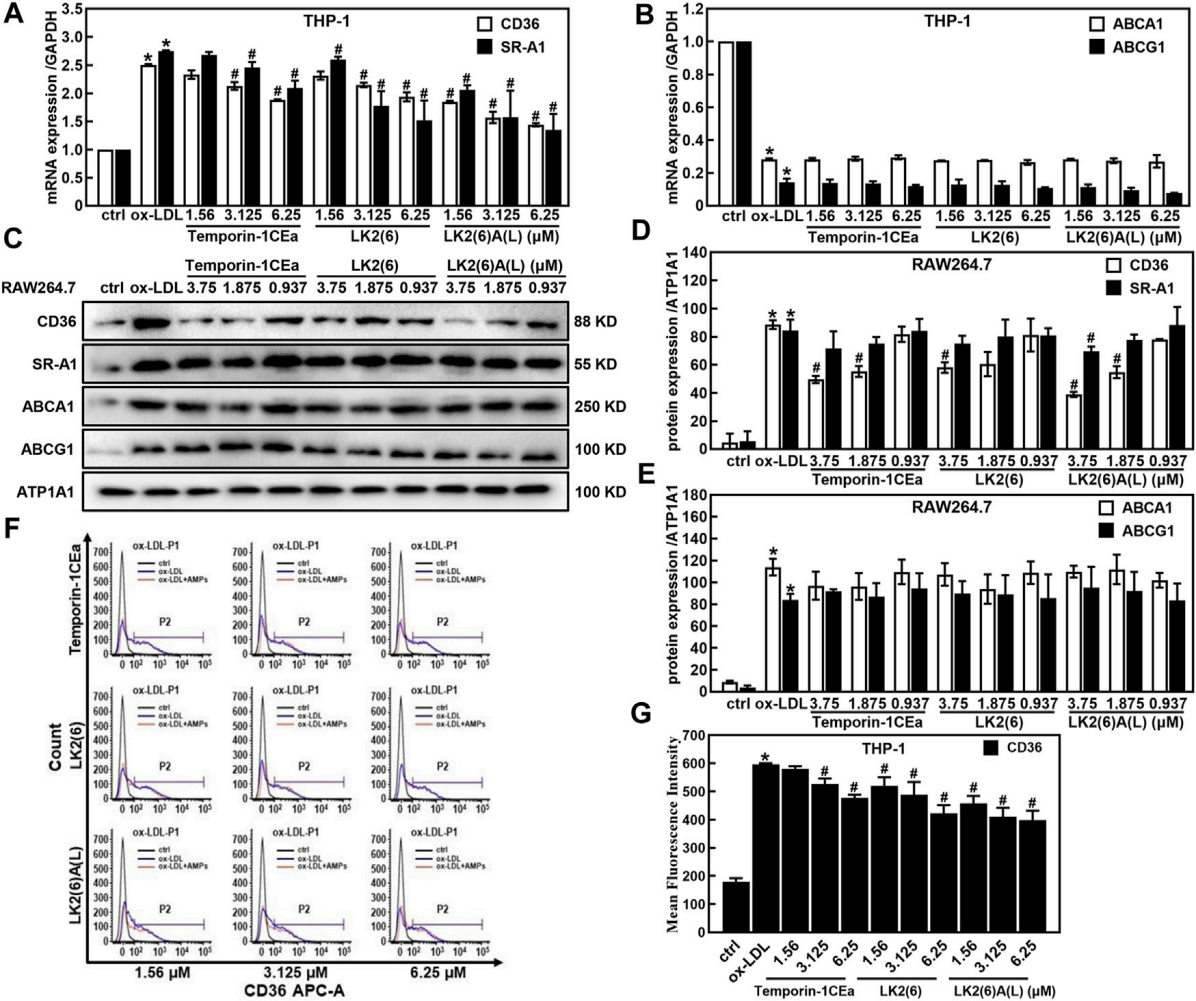
Effects of frog skin AMPs on lipid metabolism in ox-LDL induced foam cells. Gene expression of membrane proteins involved in ox-LDL uptake and cholesterol efflux by real-time quantitative PCR in foaming THP-1 cells **(A,B)** and GAPDH was used as an internal reference; Expression of proteins involved in ox-LDL uptake and cholesterol efflux by Western blot in foaming RAW264.7 cells **(C–E)** and ATP1A1 was used as an internal reference; Flow cytometry analysis of CD36 expression in foaming THP-1 cells **(F,G)**. *p < 0.05 vs. the ctrl group. ^#^ *p* < 0.05 vs. the ox-LDL group.

Accumulation of cholesterols in macrophages is because of an imbalance between the uptake and efflux of lipids. Here, to investigate the effects of temporin-1CEa and its analogs on the cellular accumulation of cholesterols, the expression of CD36 and scavenger receptors A1 (SR-A1), which are pattern-recognition receptors for ox-LDL uptake in membrane surface receptors and ATP binding cassette subfamily A/G member 1 (ABCA1/ABCG1) which are critical proteins in the extracellular efflux of lipid droplets was examined by real-time quantitative PCR, flow cytometry analysis and Western blot. As shown in Figures 3A, B, temporin-1CEa and its analogs downregulated the gene expression of CD36 and SR-A1 in a dose-dependent manner but had no effect on the gene expression of ABCA1 and ABCG1 compared to the ox-LDL group. The results show that frog skin AMPs decreased the expression of CD36 and SR-A1 by 22.6%–42.5% and 23.7%–50.9% at a concentration of 6.25 μM in foaming THP-1 cells, respectively. The same results were confirmed by Western blot experiments (Figures 3C–E). Flow cytometry results showed that the flow peak shifted to the right after the addition of ox-LDL, indicating that ox-LDL induced the expression of CD36 in THP-1 cells. After being treated with three different concentrations of temporin-1CEa and its analogs, the peak shifted to the left, and CD36 expression decreased in a concentration-dependent manner, indicating that CD36 expression was inhibited (Figures 3F, G). The results suggested that temporin-1CEa and its analogs reduced ox-LDL uptake by downregulating the protein expression of CD36 in ox-LDL-induced foam macrophages.

### Frog skin AMPs alleviated the inflammatory response induced by ox-LDL in foaming macrophages

**FIGURE 4:**
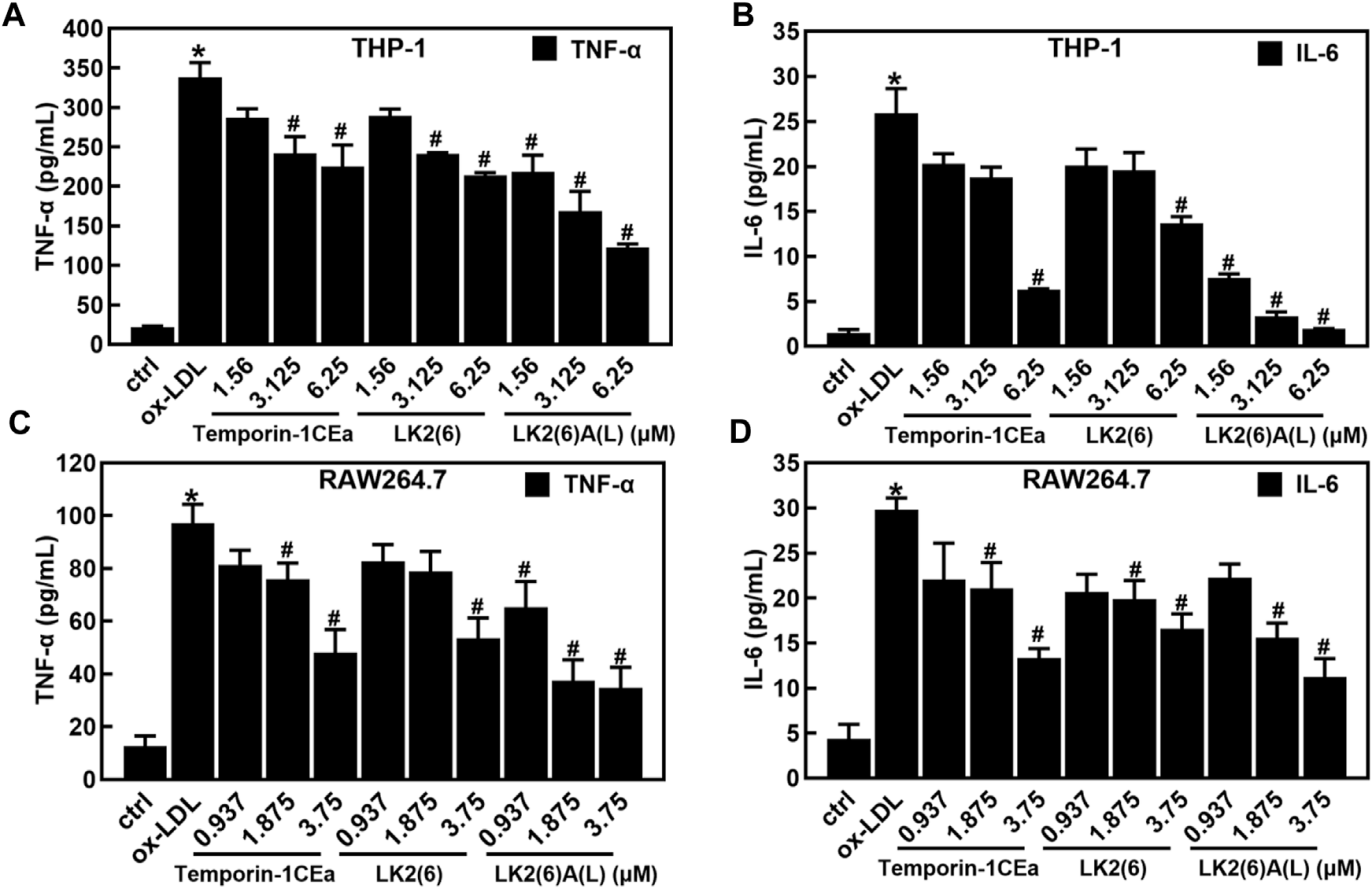
Effects of frog skin AMPs on the inflammatory response in ox-LDL induced foam cells. Levels of TNF-α and IL-6 in foaming THP-1 cells **(A,B)**; Levels of TNF-α and IL-6 in foaming RAW264.7 cells **(C,D)**. *p < 0.05 vs. the ctrl group. ^#^ *p* < 0.05 vs. the ox-LDL group.

**FIGURE 5:**
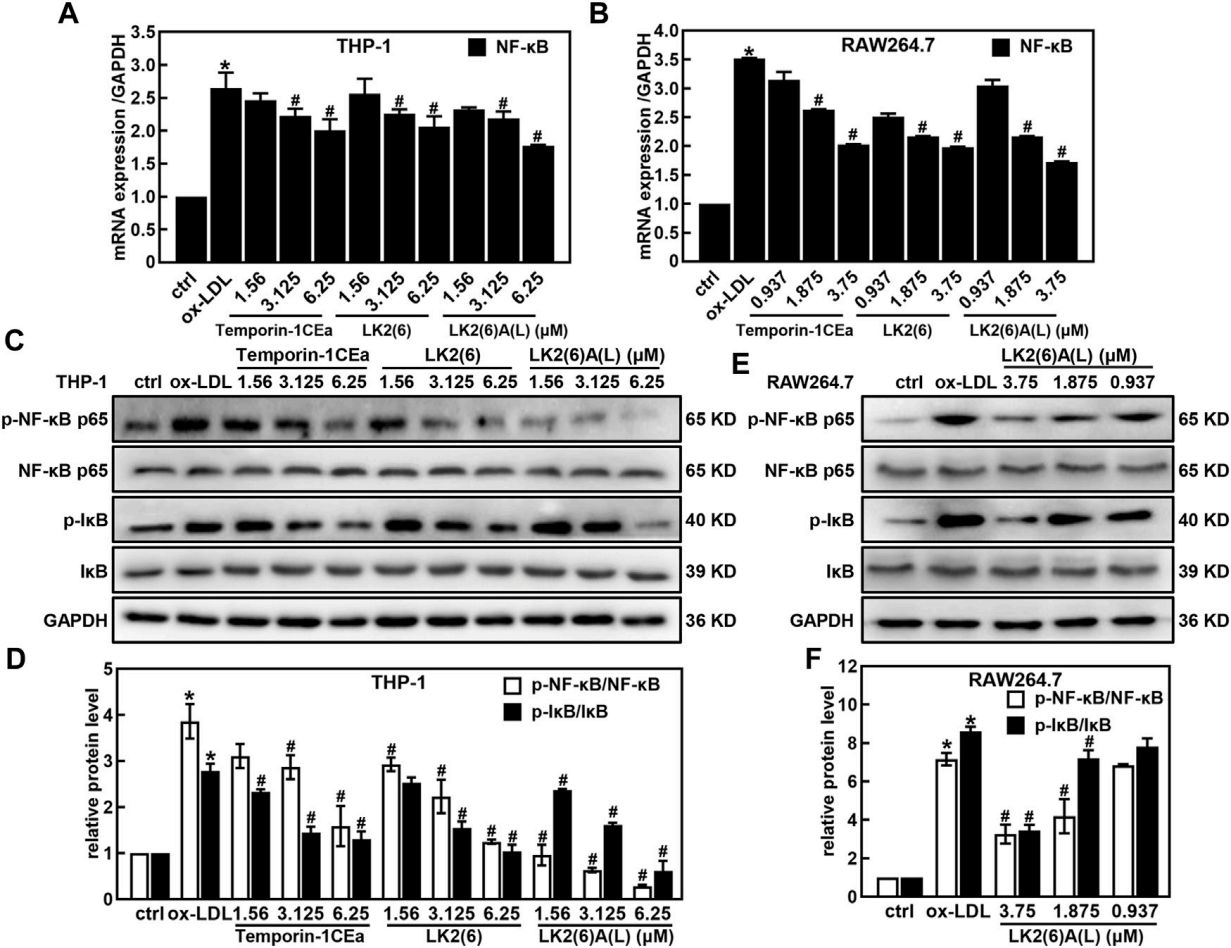
Effects of frog skin AMPs on NF-κB signaling pathway in ox-LDL induced foam cells. mRNA expression of NF-κB in the foaming THP-1 and RAW264.7 cells by real-time quantitative PCR **(A,B)**; Expression of *p-*NF-κB p65, NF-κB p65, *p-*IκB and IκB protein in the foaming THP-1 cells treated with temporin-1CEa and its analogs by Western blot **(C,D)**; Expression of *p-*NF-κB, NF-κB, *p-*IκB and IκB protein in the foaming RAW264.7 cells treated with LK2(6)A(L) by Western blot **(E,F)** and GAPDH was used as an internal reference. *p < 0.05 vs. the ctrl group. ^#^ *p* < 0.05 vs. the ox-LDL group.

As shown in Figures 4A–D, ox-LDL induced the inflammatory response in THP-1 and RAW264.7 cells, as a fact that the secretion of pro-inflammatory cytokines TNF-α and IL-6 significantly increased after cells were treated with 100 μg/mL ox-LDL. Compared to the ox-LDL group, frog skin AMPs decreased the expression of TNF-α in a dose-dependent manner by 33.4%–63.6% and 44.8%–64.2%, and the expression of IL-6 by 47.2%–92.5% and 44.4%–62.4% at a concentration of 6.25 μM in THP-1 foam cells and 3.75 μM in RAW264.7 foam cells, respectively. Studies have shown that the NF-κB and MAPK pathways are important mediators of pro-inflammatory signals from the cell surface receptor to the nucleus during the inflammatory responses of macrophages (Jayawardena et al., 2020). The results of this study showed that the mRNA expression levels of NF-κB p65 were downregulated in a concentration-dependent manner in foam cells treated with temporin-1CEa and its analogs (Figures 5A, B). LK2(6)A(L) was selected to detect the protein expressions of *p-*NF-κB p65 and *p-*IκB in ox-LDL-induced foaming cells by Western blotting in RAW264.7 cells. Consistent with the real-time qPCR results, the phosphorylated levels of NF-κB p65 and IκB were decreased in a concentration-dependent manner in both THP-1 and RAW264.7 foaming cells (Figures 5C–F). LK2(6)A(L) showed the best effects compared to temporin-1CEa and LK2(6) and reduced the proteins expression of *p*-NF-κB p65 and *p*-IκB, and the inhibition rate was more than 89.9% and 76.3% at a concentration of 6.25 μM in THP-1 foam cells, and 53.9% and 54.2% at a concentration of 3.75 μM in RAW264.7 foam cells, respectively.

**FIGURE 6:**
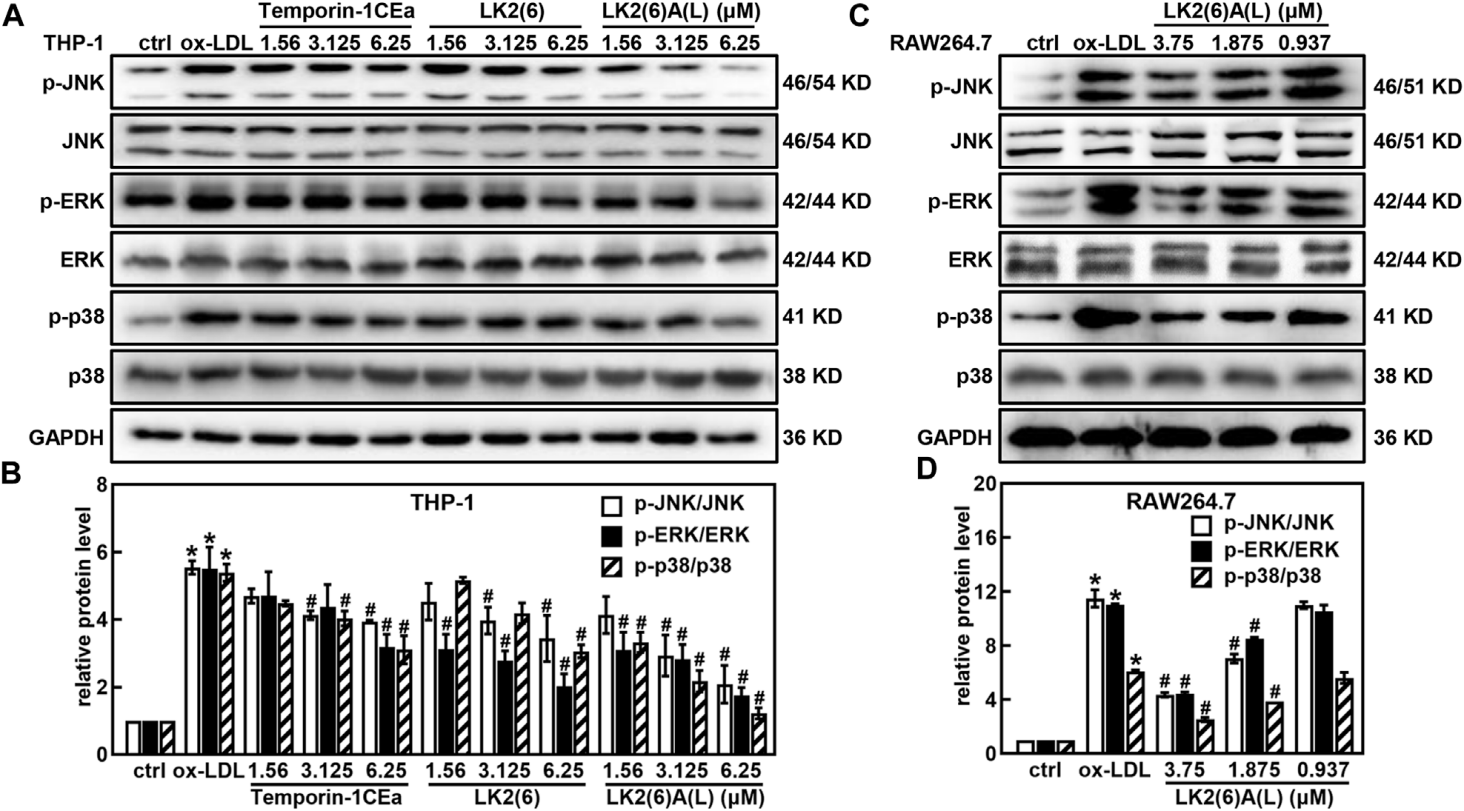
Effects of frog skin AMPs on the protein expression of MAPK signaling pathway in ox-LDL induced foam cells. Expression of *p-*JNK, JNK, *p-*ERK, ERK, *p-*p38 and p38 protein in the foaming THP-1 cells treated with temporin-1CEa and its analogs by Western blot **(A,B)**; Expression of *p-*JNK, JNK, *p-*ERK, ERK, *p-*p38 and p38 protein in the foaming RAW264.7 cells treated with LK2(6)A(L) by Western blot **(C,D)** and GAPDH was used as an internal reference. *p < 0.05 vs. the ctrl group. ^#^ *p* < 0.05 vs. the ox-LDL group.

Western blotting revealed that temporin-1CEa and its analogs reduced the protein expression of *p-*JNK, *p-*ERK and *p-*p38 in a concentration-dependent manner in ox-LDL-induced foaming THP-1 cells (Figures 6A, B). LK2(6)A(L) at a concentration of 6.25 μM reduced the expression of *p-*JNK, *p-*ERK and *p-*p38 by 71.0%, 69.5% and 74.6%, respectively. Similarly, the phosphorylated levels of JNK, ERK and p38 were decreased in a concentration-dependent manner in ox-LDL-induced foaming RAW264.7 cells treated with LK2(6)A(L). 3.75 μM LK2(6)A(L) reduced the expression of *p-*JNK, *p-*ERK and *p-*p38 by 43.5%, 47.9% and 53.8%, respectively (Figures 6C, D).

## Discussion

AS is a chronic cardiovascular disease that endangers human health and a multifactorial disease that is influenced by multiple environmental and genetic factors (Jiang et al., 2020). In the early stage of AS, LDL across the vascular endothelium by passive transport or receptor-mediated transcytosis and retained in the arterial wall. In the subendothelial space, LDL is oxidized to ox-LDL (Sima et al., 2009). Ox-LDL accelerates the development of AS. Subsequently, endothelial cells and smooth muscle cells are recruited and activated, and various cytokines and chemokines released stimulate monocytes enter the endothelium and transform into macrophages (Gupta and Sarangi, 2022). Macrophages with increased expression of scavenger receptors take up cardiovascular risk factors ox-LDL, then cholesteryl esters accumulate in macrophages and vascular cells, forming foaming cells and atherosclerotic lesions, eventually (Ma et al., 2018).

Antimicrobial peptides (AMPs) are an important part of innate immunity and exhibit broad-spectrum antibacterial activity against a wide range of microorganisms, as well as anti-inflammatory and antitumor activity (Li, 2009; Mohanty et al., 2013; Sun and Shang, 2015; Dong et al., 2018). In recent years, AMPs have attracted worldwide attention due to their potential use in the pharmaceutical and biotechnology industries (da Costa et al., 2015). Temporin-1CEa is a natural AMP that contains 17 amino acids with 4 net positive charges and an amphiphilic α-helical structure. Through substitution of amino acids, we increase the cationicity of temporin-1CEa to get LK2(6). Based on LK2(6), the hydrophobicity was increased by amino acid substitution, and LK2(6)A(L) was obtained. In the present study, we first established the macrophage foaming cells model including THP-1 derived human macrophages and murine macrophages RAW264.7 cells induced by ox-LDL. In this model, foaming cells became increasingly enlarged, and intracellular lipid droplets, total cholesterol and cholesteryl ester increased after the addition of ox-LDL in THP-1 derived human macrophages and murine macrophage RAW264.7 cells, respectively. But low concentrations of temporin-1CEa and its analogs reduced the formation of intracellular lipid droplets and the levels of TC and CE in foam cells derived from ox-LDL-induced THP-1 derived human macrophages and murine macrophages RAW264.7 cells, suggesting that these antimicrobial peptides can inhibit foaming cells formation.

Foam cells formation is dependent on increased uptake of ox-LDL or reduced efflux of cholesterol in macrophages (Sanda et al., 2021). Studies have shown that the uptake of modified LDL is mediated by scavenger receptors on the cell surface, such as CD36 and SR-A1, but CD36-and SR-A1-mediated uptake is not regulated by the negative feedback associated with intracellular cholesterol (Syvaranta et al., 2014), Song et al. showed that zafirlukast can prevent foam cells formation by reducing CD36 protein expression and inhibiting the influx of cholesterol in asthma treatment (Song et al., 2020). When macrophages accumulate large amounts of cholesterol, ABCA1 and ABCG1 promote the efflux of cholesterol, phospholipids, and other substances from macrophages (Chistiakov et al., 2016). Li et al. showed that protein arginine methyltransferase 2 (PRMT2) inhibited the formation of ox-LDL-induced RAW264.7 macrophage-derived foam cells through increased ABCA1-mediated cholesterol efflux (Li Y. Y. et al., 2020). Astragalus methionine may also promote cholesterol efflux and inhibit foaming cells formation through the upregulation of ABCG1 expression (Zhao et al., 2021). Therefore, decreased expression of CD36 and SR-A1 or increased ABCA1 and ABCG1 might inhibit forming of atherogenesis. Our results showed that temporin-1CEa and its analogs significantly decreased the expression of CD36 and SR-AI but had no effect on the expression of ABCA1 and ABCG1 in foaming THP-1 cells. There was no significant difference in the expression of SR-A1 in foaming RAW264.7 cells, and the remaining results were consistent with THP-1 cells. The above results indicated that temporin-1CEa and its analogs mainly reduced ox-LDL uptake by inhibiting CD36 expression in foaming macrophage.

Ox-LDL can induce the expression of PPARγ in macrophages, which activates the downstream target gene CD36, thereby further increasing the uptake of ox-LDL and aggravating the process of foam formation of macrophages (Collot-Teixeira et al., 2007). Some studies have shown that ox-LDL can upregulate ABCA1 and ABCG1 through the PPARγ-LXRα signaling pathway in murine macrophages RAW264.7 (Xu et al., 2009; Song et al., 2021). Macrophages are important immune cells in the human body, which clear ox-LDL accumulated in the sub-endothelium through the receptor CD36. In addition, macrophages are involved in cholesterol efflux and transport by transferring intracellular accumulated cholesterol to apoA-1 and HDL *via* ABCA1 and ABCG1 (Yu and Tang, 2022). Ox-LDL can also downregulate ABCA1 and ABCG1 in THP-1 derived human macrophages resulting in lipid metabolism disorder in macrophages (Wang et al., 2018; Cai et al., 2022). These results are consistent with the results of our study.

Inflammation is a well-established risk factor for AS (Kong et al., 2022). Ox-LDL can activate macrophages, make them differentiate into M1 inflammatory type, and trigger inflammatory response, leading to the continuous release of inflammatory factors (Bhattacharya et al., 2022). Foam cells can trigger inflammatory responses, promoting the development of AS through synergistic effects (Li T. et al., 2020). The mechanisms that link inflammatory responses to lipid deposition in macrophages have not yet been defined. Ox-LDL induces inflammatory response in foam cell and the release of inflammatory factors such as TNF-α and IL-6 *via* the NF-κB and MAPK signaling pathways (Wang et al., 2020; Zhang et al., 2020). NF-κB normally binds with IκB in the cytoplasm, preventing the entry of NF-κB into the nucleus. Phosphorylation of the IκB kinase (IKK) complex and NF-κB activation leads to high expression of inflammatory factors such as TNF-α and IL-6 (Sun et al., 2017; Huang et al., 2019). Previous studies have suggested that NF-κB nuclear localization sequence (NLS) peptide can targeting NF-κB nuclear translocation hampers inflammation and atherosclerosis development (Mallavia et al., 2013). Free cholesterol in foam macrophages was increased and leads to the induction and secretion of two inflammatory cytokines TNF-α and IL-6. The increases in TNF-α and IL-6 mRNA and protein were mediated by free cholesterol-induced activation of the IκB/NF-κB signaling pathway (Li et al., 2005).

Likewise, MAPK signaling pathway is found to mediate extracellular signaling and cellular and nuclear responses (Simion et al., 2020; Zhao et al., 2020). Some drugs, such as Geniposide and ginsenoside compound K, through the MAPK signaling pathway, attenuate ox-LDL induced macrophage foaming and inflammation (Jin et al., 2020; Lu et al., 2020). The MAPK signaling pathway is one of the important pathways in the biological signal transduction network, and it is the key signal pathway of cellular inflammatory response. MAPK is an evolutionarily conserved group of serine-threonine kinases that can be divided into JNK, ERK and p38 MAPK signaling pathways. MAPK signaling pathway is a cascade of phosphorylation (Reustle and Torzewski, 2018). The JNK signaling pathways play important roles in stress responses such as inflammation and apoptosis, and can be activated by the cytokine TNF-α (Wullaert et al., 2006; Reustle and Torzewski, 2018). Heat shock protein 70 (HSP70) accelerates atherosclerosis by downregulating the expression of ABCA1 and ABCG1 through the JNK signaling pathway (Sharapova et al., 2021). ERK is integral to the uptake of ox-LDL by human macrophages (Li et al., 2010). Resveratrol and Tribulus terrestris L. extract ameliorates atherosclerosis by inhibition of vascular smooth muscle cell proliferation in ApoE^−/−^ mice *via* suppression of ERK signaling pathway (Guo S. et al., 2022; Zhang et al., 2022). P38 MAPK signaling pathway activation is associated with the release of inflammatory cytokines, which also activates p38 MAPK in turn (Voutyritsa et al., 2021). The development of AS tends to activate the MAPK signaling pathway, and geniposide can reduce LPA-induced RAW264.7 macrophage-derived foam cells formation through the p38 MAPK signaling pathway (Shen et al., 2019). Increased mRNA and protein expression of inflammatory factors such as TNF-α and IL-6 in foam cells was induced by FC, which mediated the activation of JNK1/2, ERK1/2 and p38 MAPK signaling pathways (Li et al., 2005). In our study, frog skin peptide temporin-1CEa and its analogs reduced the expression of THP-1 derived human macrophages and murine macrophage RAW264.7 cytokines TNF-α and IL-6 in a dose-dependent manner. All the three frog skin AMPs decreased the mRNA expression of NF-κB p65 in both foam cells and the protein expression of *p*-NF-κB p65 and *p*-IκB in THP-1 cells. LK2(6)A(L) decreased the protein expression of *p*-NF-κB p65 and *p*-IκB in RAW264.7 cells. The results suggest that temporin-1CEa and its analogs exert an inhibitory effect on NF-κB signaling pathway, which in turn inhibits the release of downstream inflammatory factors TNF-α and IL-6. The frog skin AMPs also had inhibitory effects on MAPK signaling pathways, and reduced the expression of *p*-JNK, *p*-ERK and *p*-p38 proteins, among which LK2(6)A(L) had the most significant effect.

The above results suggested frog skin peptide temporin-1CEa and its analogs might regulate the uptake of ox-LDL by foam cells through CD36 and improve the accumulation of lipid droplets and cholesterol in foam cells. It can inhibit ox-LDL-induced phosphorylation of NF-κB p65 and MAPK signaling pathway components in foam-derived macrophages. However, the present study has several limitations. First, our study showed that frog skin peptide regulated lipid uptake in foam cells through CD36, but the regulation mechanism is unclear. PPARγ is an important regulator of CD36, and studies suggested that ox-LDL regulated the expression of CD36 through PPARγ (Collot-Teixeira et al., 2007), but it is unclear whether frog skin peptide plays a role in the regulation of PPARγ. Secondly, MAPK signaling pathway can regulate the inflammatory response of foam cells through PPARγ phosphorylation (Hosooka et al., 2008). The post-translational modification of PPARγ, especially phosphorylation modification, plays an important role in the lipid metabolism (Yang et al., 2022; Yin et al., 2022). The three frog skin peptides could inhibit MAPK and NF-κB signaling pathways, but it is unclear whether they regulated PPARγ phosphorylation through MAPK signaling pathway, and inhibit the inflammatory response of foam cells, which is worthy of further study. Finally, the occurrence and development of AS are accompanied by lipid metabolism disorders and inflammation. Although the mechanism by which the three frog skin peptides affect lipid metabolism and anti-inflammation of AS through NF-κB and MAPK signaling pathways is still not fully elucidated, temporin-1CEa and its analogs provide new candidate therapeutic drugs for the treatment of AS, which need to be further studied.

## Data Availability

The datasets presented in this study can be found in online repositories. The names of the repository/repositories and accession number (s) can be found in the article/Supplementary Material.
