# Supplementary material for: Effects of frog skin peptide temporin-1CEa and its analogs on ox-LDL induced macrophage-derived foam cells
Source: Front Pharmacol. 2023 Mar 20;14:1139532. doi: 10.3389/fphar.2023.1139532 (PMC10067733; doi:10.3389/fphar.2023.1139532)
Supplement: Supplementary file 1 [file Table2.DOCX]

Raw Data:

https://www.jianguoyun.com/p/DaE4npEQub7RCBiooq4EIAA
